# Inorganic Nitrate in Angina Study: A Randomized Double‐Blind Placebo‐Controlled Trial

**DOI:** 10.1161/JAHA.117.006478

**Published:** 2017-09-08

**Authors:** Konstantin Schwarz, Satnam Singh, Satish K. Parasuraman, Amelia Rudd, Lee Shepstone, Martin Feelisch, Magdalena Minnion, Shakil Ahmad, Melanie Madhani, John Horowitz, Dana K. Dawson, Michael P. Frenneaux

**Affiliations:** ^1^ School of Medicine & Dentistry University of Aberdeen Aberdeen UK; ^2^ Royal Wolverhampton Hospital Wolverhampton UK; ^3^ Norwich Medical School University of East Anglia Norwich UK; ^4^ University of Southampton Southampton UK; ^5^ Aston Medical Research Institute Aston University Birmingham UK; ^6^ Basil Hetzel Institute University of Adelaide Adelaide Australia; ^7^ Institute of Cardiovascular Sciences University of Birmingham Birmingham UK

**Keywords:** angina, exercise, inorganic nitrate, ischemia, nitrite, randomized trial, Ischemia

## Abstract

**Background:**

In this double‐blind randomized placebo‐controlled crossover trial, we investigated whether oral sodium nitrate, when added to existing background medication, reduces exertional ischemia in patients with angina.

**Methods and Results:**

Seventy patients with stable angina, positive electrocardiogram treadmill test, and either angiographic or functional test evidence of significant ischemic heart disease were randomized to receive oral treatment with either placebo or sodium nitrate (600 mg; 7 mmol) for 7 to 10 days, followed by a 2‐week washout period before crossing over to the other treatment (n=34 placebo‐nitrate, n=36 nitrate‐placebo). At baseline and at the end of each treatment, patients underwent modified Bruce electrocardiogram treadmill test, modified Seattle Questionnaire, and subgroups were investigated with dobutamine stress, echocardiogram, and blood tests. The primary outcome was time to 1 mm ST depression on electrocardiogram treadmill test. Compared with placebo, inorganic nitrate treatment tended to increase the primary outcome exercise time to 1 mm ST segment depression (645.6 [603.1, 688.0] seconds versus 661.2 [6183, 704.0] seconds, *P*=0.10) and significantly increased total exercise time (744.4 [702.4, 786.4] seconds versus 760.9 [719.5, 802.2] seconds, *P*=0.04; mean [95% confidence interval]). Nitrate treatment robustly increased plasma nitrate (18.3 [15.2, 21.5] versus 297.6 [218.4, 376.8] μmol/L, *P*<0.0001) and almost doubled circulating nitrite concentrations (346 [285, 405] versus 552 [398, 706] nmol/L, *P*=0.003; placebo versus nitrate treatment). Other secondary outcomes were not significantly altered by the intervention. Patients on antacid medication appeared to benefit less from nitrate supplementation.

**Conclusions:**

Sodium nitrate treatment may confer a modest exercise capacity benefit in patients with chronic angina who are taking other background medication.

**Clinical Trial Registration:**

URL: https://www.clinicaltrials.gov/. Unique identifier: NCT02078921. EudraCT number: 2012‐000196‐17.

**Clinical Perspective**

**What Is New?**

- In patients suffering from chronic angina, sodium nitrate treatment appears to have a modest anti‐ischemic effect when added to other background medication.
- We report a trend toward improved myocardial ischemia and significantly increased exercise capacity.
- Patients with stomach acid suppression appeared less likely to benefit, and mild nausea and vomiting were rarely reported.

**What Are the Clinical Implications?**

- Although there is currently not enough evidence to recommend routine sodium nitrate supplementation to patients with angina, increased dietary inorganic nitrate intake from sources such as green leafy vegetables is unlikely to cause any significant side effects, may improve exercise capacity, and can be recommended as a natural complement to other medication in patients with chronic angina.
- The treatment period was relatively short, and a longer‐term supplementation trial will need to be established in future studies.

## Introduction

Clinicians are increasingly encountering patients with diffuse coronary artery disease years after their revascularization, when repeated intervention is either impossible or of limited benefit. Approximately 30% of “completely revascularized” patients still continue to experience angina.^1^ Current antianginal drugs are very effective, but their use can be precluded because of side effects (especially bradycardia or hypotension).

Experimental evidence suggests that targeting the nitrate‐nitrite‐nitric oxide pathway may have therapeutic potential in patients with angina. Plasma nitrite is derived both from oxidation of endothelium‐derived nitric oxide and from dietary sources via bioconversion of nitrate involving an enterosalivary circulation, reduction by the oral microbial flora, and low stomach pH.^2^

The vasodilator effect of nitrite is potentiated by hypoxia^3^, ^4^, ^5^, ^6^ and, unlike organic nitrates (eg, glyceryl trinitrate [GTN], isosorbide 5‐mononitrate, and isosorbide dinitrate), not subject to development of tolerance.^7^

Nitrate has been reported to improve metabolic efficiency during exercise in human skeletal muscle, but it is unknown whether this also occurs in cardiac muscle.^8^, ^9^, ^10^, ^11^, ^12^ Under experimental conditions both nitrite and nitrate led to protection from ischemia‐reperfusion injury,^13^, ^14^, ^15^, ^16^, ^17^ blood pressure reduction,^18^, ^19^, ^20^, ^21^ reversal of pulmonary arterial hypertension,^5^, ^22^, ^23^ and induction of angiogenesis.^24^, ^25^, ^26^

In light of these preclinical experimental results we hypothesized that oral sodium nitrate supplementation will delay the onset of myocardial ischemia in patients suffering from chronic angina.

## Methods

### Design

**Figure 1:**
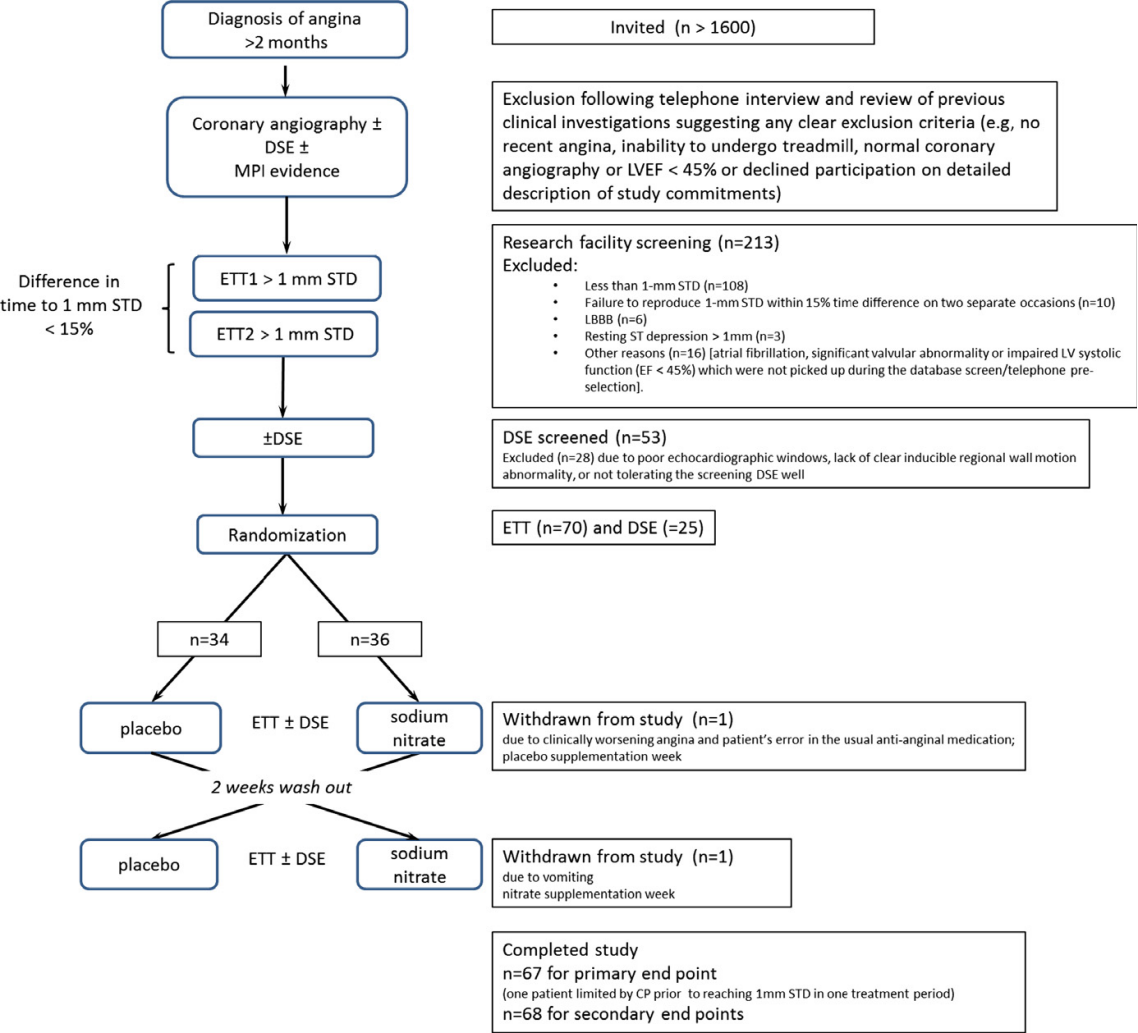
CONSORT diagram. DSE indicates dobutamine stress echocardiogram; ETT, electrocardiogram treadmill test; LBBB, left bundle branch block; LVEF, left ventricle ejection fraction; STD, ST segment depression.

This randomized double‐blind, placebo‐controlled crossover trial (Figure 1) was approved by the Scotland A Research Ethics Committee, subject to Medicines and Healthcare products Regulatory Agency regulation, and run in accordance with the Declaration of Helsinki. All patients signed an informed written consent.

### Patient Selection and Protocols

Patients aged ≥18 years with exertional angina (≥2 months duration) were recruited.

Inclusion criteria comprised a positive electrocardiogram treadmill test (ETT) and either angiographic evidence of obstructive coronary artery disease or, if not available, a positive functional test (dobutamine stress echocardiogram [DSE] or myocardial perfusion scan). Exclusion criteria were inability to perform ETT, significant valvular disease, nonsinus rhythm, women of childbearing potential, G6PD (glucose‐6‐phosphate dehydrogenase) deficiency, LV ejection fraction <45% or New York Heart Association heart failure class III or IV, myocardial infarction or coronary revascularization within the past 2 months, resting ST segment depression ≥1 mm, or left bundle branch block.

Patients were screened with modified Bruce‐protocol ETTs on separate days and enrolled only if they had replicable difference (≤15%) in time to 1‐mm ST segment depression between the first and the second screening.^27^

Patients continued regular medications including antianginal agents at a fixed dose with the exception of long‐acting organic nitrates, which were stopped at least 72 hours before enrollment. The latter served to avoid potential interaction between organic and inorganic nitrates, which partially act via similar downstream pathways and may confound the outcome. The use of short‐acting sublingual GTN was allowed. Some patients undergoing a concomitant DSE were asked to omit their β‐blocker for 48 hours before their visits (to facilitate their dobutamine response) at the discretion of the researcher, mainly depending on symptom severity, and the elected strategy was used consistently throughout the screening and all subsequent visits. This approach was permissible due to the crossover design in which each patient served as his or her own control.

### Study Outcomes

The primary outcome was the time to a 1‐mm ST segment depression on ETT.

Secondary outcomes were time to chest pain onset, total exercise time, angina and GTN use frequency, modified Seattle Questionnaire, nitrate and nitrite plasma levels, angiogenic markers, and myocardial contractility assessment by peak systolic velocity measured by Doppler imaging (details described elsewhere).^28^

### Intervention and Randomization Process

The trial medication was manufactured and placed into packs containing 2 bottles labeled 1 and 2 at the Western Glasgow Infirmary Pharmacy. Each bottle contained 14 capsules with either 600 mg (7 mmol) of sodium nitrate or placebo (lactose monohydrate).

Researchers and patients were blinded to the randomization sequence in individual packs. Patients started treatment with bottle 1, 1 capsule a day for a period of 7 to 10 days, before undergoing a treadmill test and/or DSE and/or blood tests. After a 2‐week washout period the same procedure was repeated with bottle 2. Compliance with medication was assessed by patient via a checklist and by an investigator calculating the remaining capsules from return bottles.^28^ Patients were asked to follow a low‐nitrate and ‐nitrite diet, limit caffeine intake, and avoid use of antibacterial mouthwash.^29^ The 600‐mg sodium nitrate dose was based on previous evidence that 300 mg to 600 mg of oral nitrate was effective in multiple studies assessing effects on blood pressure and exercise capacity in human volunteers.^10^, ^11^, ^20^, ^21^, ^30^

### Exercise Treadmill Test

Four ETTs, 2 screening and 1 following each treatment period, were performed at the same time of day, in an air‐conditioned room (21°C), ≈2 hours following ingestion of the last study capsule, on a CASE Exercise Testing System (GE Healthcare, Chicago, IL). Automated blood pressure (BP) (Tango, SunTech Medical, Morrisville, NC) and 12‐lead ECGs were recorded in a standing position before exercise, at the end of each 3‐minute stage, at the time of first 1‐mm ST depression, at the time of first chest pain onset, at peak exercise, and every 3 minutes into recovery. In patients with minor resting ST depression (<1 mm), the time to 1‐mm ST change was defined as additional ST depression of 1 mm below the resting value.^31^ A 1‐mm ST depression was digitally detected and displayed by the CASE software at the J point (+80 milliseconds).^28^

### Dobutamine Stress Echocardiography

All patients were invited to participate in the DSE subgroup. Details of the full protocol are provided elsewhere.^28^ In brief, patients with inducible regional wall motion abnormalities and satisfactory echo windows who tolerated the baseline scan were enrolled. Exams were performed on a Vivid 9 machine (GE Healthcare) ≈2 hours after completion of the treadmill test. The protocol involved acquisition at rest, baseline, and the following stages with incremental dobutamine dose: 10, 20, 30, and 40 μg/kg per minute. For the screening test we used left ventricle contrast (Sonovue, Bracco Imaging, Milan, Italy) in order to improve sensitivity of detection of inducible regional wall abnormalities.^32^ For on‐treatment tests, images were obtained without contrast using Doppler tissue velocity imaging in apical views and later analyzed using Echo PAC (version 1.113; GE Healthcare). Longitudinal basal segment peak systolic velocity was measured in 6 basal segments as previously described.^33^ The percentage increase in peak systolic velocity at peak dobutamine stage versus baseline was used for the final analysis. Differences in global and ischemic segment only (as defined on screening contrast test) were analyzed.^28^

### Blood Tests, Modified Seattle Questionnaire, GTN Use, and Angina Frequency

A subgroup of 20 patients underwent blood tests for nitrate and nitrite levels and angiogenic markers (soluble fms‐like tyrosine kinase receptor‐1, placental growth ractor, and vascular endothelial growth factor) levels. Samples were collected and analyzed as previously reported.^28^, ^34^ We modified the Seattle Questionnaire^35^ to reflect the short treatment period of 1 week when compared to the original assessment over 4 weeks. Frequency of angina attacks and GTN use were documented on a checklist.

### Statistical Analysis

In previous randomized studies of add‐on antianginal medication (amlodipine, organic nitrates, atenolol, ranolazine, ivabradine, or allopurinol)^27^, ^36^, ^37^, ^38^, ^39^ the mean difference in time to 1‐mm ST depression between the active and placebo groups was ≈50 seconds. The SD in crossover studies was ≈80 to 90 seconds.^36^, ^40^, ^41^, ^42^ Based on a mean treatment difference of 30 seconds and a SD of 80 seconds with a type I error of 5% and statistical power 80%, we would require 58 patients. To allow for dropouts we planned to randomize 70 patients. We sought to recruit a minimum of 20 patients for the secondary end point of DSE peak systolic velocity, and its sample sizing is described elsewhere.^28^

The within‐patient differences in primary and secondary efficacy end points were assumed to follow a normal distribution. The analysis followed recommendations by Senn^43^ for a 2‐treatment and 2‐period crossover trial analysis. A general linear model was constructed with the following factors included: patient (as a random effect), period, and treatment (both as fixed effects). Treatment efficacy was estimated as the treatment effect estimate from the model with a 95% confidence interval and the hypothesis of 0 effect tested (5% significance level). An interaction term between treatment and period was included to assess the possibility of a carryover effect. Where this was statistically significant (*P*<0.05), the treatment effect estimate was also provided with this interaction included in the model. The subgroup analysis (which was not defined before the analysis) was carried out in patients not on proton pump inhibitors or H_2_ blockers. The assumption of a normal distribution was checked via a graphic inspection of the residuals. All analyses were carried out in SAS version 9.3 (SAS Institute, Cary, NC) by a trial statistician who conducted and reported the analyses subgroup blind.

## Results

### Recruitment and Baseline Characteristics

More than 1600 patients with a diagnosis of angina were invited to participate. Following telephone or clinic interview only 213 potentially suitable patients gave informed consent and were invited for detailed screening investigations. Of these 70 patients were found to be eligible and were randomized (Figure 1, Table 1).^44^ With overall 93.6% of patients taking all their capsules correctly as prescribed (131 out of 140 treatment periods) the compliance was high. All patients attending for their final day visit took their last capsules in the morning as planned (Data S1 and Table S1).

**Table 1:** Demographics

Mean (SD) or n (%)	All Subjects	Nitrate First (n=36)	Placebo First (n=34)
Age, y	67.3 (7.7)	66.5 (7.9)	68.3 (7.7)
Height, cm	168.0 (8.3)	169.8 (8.1)	166.2 (8.3)
Weight, kg	80.8 (13.6)	84.0 (14.6)	77.5 (11.9)
BMI	28.6 (4.0)	29.1 (4.6)	28.0 (3.4)
LVEF (%)	59.5 (7.1)	59.8 (6.8)	59.2 (7.5)
Systolic BP, mm Hg	140.1 (18.1)	137.2 (17.9)	143.3 (18.1)
Diastolic BP, mm Hg	79.4 (10.2)	78.9 (10.7)	80.0 (10.0)
Heart rate, bpm	61.9 (12.3)	60.8 (12.7)	63.2 (12.0)
Sex			
Male	52 (74%)	31 (86%)	21 (62%)
Female	18 (26%)	5 (14%)	13 (38%)
CCS class^a^			
1	29 (41%)	16 (44%)	13 (38%)
2	34 (49%)	17 (47%)	17 (50%)
3	7 (10%)	3 (9%)	4 (12%)
4	0	0	0
Ischemia test			
Angio only	29 (41%)	16 (44%)	13 (38%)
Angio and DSE	30 (43%)	14 (39%)	16 (47%)
Angio, DSE, and MPI	3 (4%)	0	3 (9%)
Angio and MPI	2 (3%)	1 (3%)	1 (3%)
DSE only	6 (9%)	5 (14%)	1 (3%)
Vessel disease			
Single vessel	23 (33%)	10 (28%)	13 (38%)
2 vessels	13 (19%)	8 (22%)	5 (15%)
3 vessels	12 (17%)	7 (19%)	5 (15%)
Residual disease^b^	17 (24%)	7 (19%)	10 (29%)
No angiography	5 (7%)	4 (11%)	1 (3%)
Previous MI	29 (41%)	13 (36%)	16 (47%)
HTN	36 (51%)	19 (53%)	17 (50%)
DM	21 (30%)	7 (19%)	14 (41%)
PAD	12 (17%)	6 (17%)	6 (18%)
Stroke or TIA	6 (9%)	2 (6%)	4 (12%)
Smoker			
Never	32 (46%)	18 (50%)	14 (41%)
Ex‐smoker	35 (51%)	17 (47%)	18 (53%)
Current	2 (3%)	1 (3%)	1 (3%)
Missing	1 (1%)		1 (3%)
Previous revascularization			
None	39 (56%)	24 (67%)	15 (44%)
PCI	16 (23%)	5 (14%)	11 (32%)
CABG	8 (11%)	4 (11%)	4 (12%)
PCI and CABG	7 (10%)	3 (8%)	4 (12%)
Baseline medication			
Aspirin	67 (96%)	34 (94%)	33 (97%)
β‐Blocker	52 (74%)^c^	29 (81%)	23 (68%)
Long‐acting organic nitrate	27 (39%)^d^	15 (42%)	12 (35%)
Ivabridine	2 (3%)	0	2 (6%)
Calcium channel blocker	24 (34%)	11 (31%)	13 (38%)
Nicorandil	13 (19%)	4 (11%)	9 (26%)
Statin	62 (89%)	31 (86%)	31 (91%)
ACE‐I	25 (36%)	16 (44%)	9 (26%)
ARB	13 (19%)	3 (8%)	10 (29%)
PPI	24 (34%)	12 (33%)	12 (35%)
H_2_ blocker	3 (4%)	1 (3%)	2 (6%)
Other	60 (86%)	33 (92%)	27 (79%)

ACE‐I indicates angiotensin‐converting enzyme inhibitor; ARB, angiotensin receptor blocker; BMI, body mass index; BP, blood pressure; CABG, coronary artery bypass graft; DM, diabetes mellitus; HTN, hypertension; LVEF, left ventricle ejection fraction; MI, myocardial infarction; PAD, peripheral artery disease; PCI, percutaneous coronary intervention; PPI, proton pump inhibitor; TIA, transient ischemic attack.

a Canadian Cardiovascular Society Classification of angina severity^44^.

b Previously revascularized multivessel disease with at least single‐vessel residual disease at time of angiography but unable to rule out progression of disease by the time of study entrance.

c β‐Blockers were omitted in selected patients (18 [26%]) for 48 hours prior each visit to facilitate dobutamine stress echocardiography; uninterrupted β‐blocker treatment continued in 34 (49%) patients.

d Oral long‐acting nitrates were stopped before randomization in all participants.

### Electrocardiogram Treadmill Test

**Figure 2:**
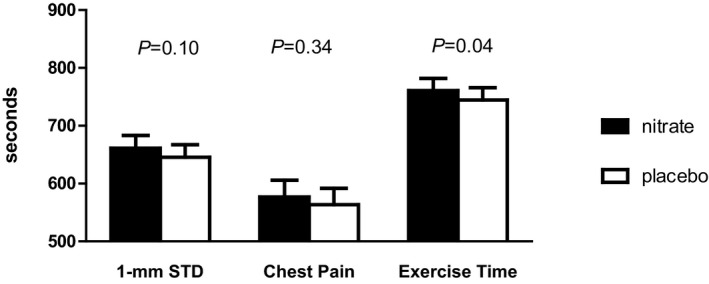
Exercise treadmill test. Columns display mean±SEM time until either STD or chest pain and duration of exercise. STD indicates ST segment depression.

The time from the last nitrate capsule ingestion to start of the treadmill test was 154.0 (139.5‐169.3) minutes (median [interquartile range]). During the nitrate treatment, relative to placebo, there were trends toward reduction of various manifestations of ischemia including time to 1‐mm ST depression, time to onset of chest pain, and total exercise time (Figure 2). However, only changes in total exercise time were statistically significant (median [95% confidence interval] 760.9 [719.5, 802.2] versus 744.4 [702.4, 786.4] seconds, *P*=0.04), whereas for time to 1‐mm ST depression (661.2 [618.3, 704.0] versus 645.6 [603.1, 688.0] seconds, nitrate versus placebo, *P*=0.10). There was a significant treatment and period interaction effect with respect to total exercise time, but inclusion of this interaction term made no material difference to the treatment effect estimate.

Post hoc subgroup analysis of the primary end point in patients not on proton pump inhibitors or H_2_ blockers (n=43) revealed a near‐significant increased time to 1‐mm ST depression in the nitrate arm (estimated effect size +21.89 seconds, *P*=0.070) (Table 2).

**Table 2:** Efficacy End Points

	N	Nitrate Mean (SD)	Placebo Mean (SD)	Difference Mean (SD)	Effect Estimate^a^ (95% CI)	*P* Value
Time to 1‐mm ST depression (s)	67^b^	661.2 (179.0)	645.6 (177.2)	15.6 (80.9)	16.21 (−3.4 to 35.8)	0.104
Time to chest pain (s)	49	576.9 (201.5)	563.5 (197.9)	13.4 (98.1)	13.56 (−14.7 to 41.8)	0.343
Total exercise time (s)	67^b^	760.9 (172.7)	744.4 (175.4)	16.5 (69.5)	17.53 (0.6‐34.3) 18.33 (1.5‐35.2)	0.041 0.033
Global peak systolic velocity (% increase)	25	73.11 (33.1)	72.6 (31.7)	0.43 (31.0)	−0.23 (−13.1 to 12.6)	0.972
Ischemic segment peak systolic velocity (% increase)	24	64.9 (43.4)	60.8 (36.4)	4.08 (29.2)	5.14 (−15.5 to 25.8)	0.623
Seattle questionnaire score	64	101.8 (11.2)	102.7 (10.9)	−0.9 (8.6)	−0.90 (−3.0 to 1.2)	0.406
Angina attack episodes	67	1.2 (2.5)	1.1 (2.2)	0.07 (1.4)	0.07 (−0.30 to 0.43) 0.06 (−0.30 to 0.43)	0.712 0.730
GTN use	67	0.6 (1.7)	0.5 (1.4)	0.09 (1.1)	0.09 (−0.1 to 0.3) 0.09 (−0.18 to 0.37)	0.514 0.490
Not taking PPI or H_2_ receptor blockers						
Time to 1‐mm ST depression (s)	43	662.1 (174.3)	641.0 (170.6)	21.0 (76.9)	21.8 (−1.6 to 45.4)	0.070

CI indicates confidence interval; GTN, glyceryl trinitrate; PPI, proton pump inhibitor.

a From linear model including period effect and also a treatment‐period (ie, “carryover”) effect when this was found to be significant (lower figures).

b Subject 66 missed both periods (withdrawn due to medication error); subject 46 (withdrawn due to nausea), and subject 181 missing second period (limiting chest pain before reaching 1‐mm STD).

### Dobutamine Stress Echocardiography

Fifty‐three patients underwent a screening DSE, 25 of whom were enrolled into the DSE arm. Change in global systolic velocity (baseline to peak) was not significantly altered by nitrate treatment (*P*=0.972) or when only ischemic segments were analyzed (*P*=0.623) (Table 2).

### Modified Seattle Questionnaire, GTN Use, and Angina Frequency

There was no significant difference in the Modified Seattle Questionnaire score, GTN use, or in angina frequency between the treatment arms (Table 2).

### Bloods

**Figure 3:**
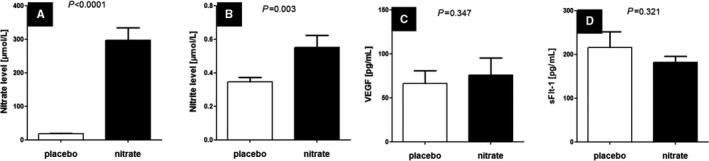
Plasma levels of (A) nitrate, (B) nitrite, (C) VEGF, and (D) sFlt‐1. Data are displayed as mean±SEM. sFlt indicates soluble fms‐like tyrosine kinase receptor; VEGF, vascular endothelial growth factor.

The time from the last nitrate capsule ingestion on the morning of the visit to the blood test was 135.0 (129.3‐157.5) minutes (median [IQR]). Compared with placebo the nitrate‐treated arm had significantly higher plasma nitrate (mean [SD] 18.3 [6.5] versus 297.6 [164.3] μmol/L, *P*<0.0001) and nitrite (mean [SD] 346 [124] versus 552 [320] nmol/L, *P*=0.003; Figure 3).

There was no significant difference in angiogenic markers between the placebo and nitrate arms (mean [SD] vascular endothelial growth factor, 66.5 [65.3] versus 76.1 [87.2] pg/mL, *P*=0.347; mean [SD] soluble fms‐like tyrosine kinase receptor‐1, 216.1 [160.4] versus 182.0 [62.2] pg/mL, 0.321; Figure 3).

### Blood Pressure

There was no difference in BP obtained at rest (nitrate versus placebo, systolic BP 132.4 [18.2] versus 131.3 [22.8] mm Hg, *P*=0.670; diastolic BP 76.3 [11.0] versus 76.9 [13.2] mm Hg, *P*=0.519) or at peak exercise (nitrate versus placebo, systolic BP 175.3 [26.0] versus 173.0 [27.4] mm Hg, *P*=0.427; diastolic BP 76.5 [12.2] versus 75.6 [12.6] mm Hg, *P*=0.626).

### Adverse Events

In general the treatment was tolerated well. Gastrointestinal side effects were more common in the nitrate arm (Table 3). One patient reported severe vomiting following the capsule intake for 3 consecutive mornings and was withdrawn from the study.

**Table 3:** Adverse Events

n (%)	Nitrate	Placebo
Nausea/abdominal cramps	6 (9%)	3 (4%)
Vomiting	3 (4%)	0
Dry mouth	1 (1%)	1 (1%)
Tiredness	1 (1%)	1 (1%)
Hot flushes	1 (1%)	1 (1%)
Headache	0	3 (4%)
Loose stool	0	1 (1%)

## Discussion

Sodium nitrate treatment added to other background medication failed to reach the predefined level of statistical significance for the difference in the primary end point (time to 1‐mm ST depression). However, there was a trend to improvement in this outcome and a statistically significant increase in the predefined secondary end point, total exercise time, supporting a modest anti‐ischemic effect. All treadmill test parameters trended to improve performance with nitrate supplementation (Figure 2). On a post hoc analysis there was a strong positive correlation among the time to 1‐mm ST depression, total exercise time, and time to chest pain onset (Pearson r varied between 0.6 and 0.7, all *P*<0.001, Figures S1 through S3).

The observed SD of difference in means of time to 1‐mm ST depression was in line with the SD used for our sample size calculations. The sample size calculations assumed a mean difference in this primary outcome measure of 30 seconds based on previous trials in the field. Although the estimate of effect was indicative of a benefit of nitrate (+16.21 [−3.4 to +35.8] seconds, effect estimate [95% confidence interval]), the difference did not reach statistical significance. This may be because there is indeed no benefit or may reflect a more modest benefit than we had assumed in our sample size calculations. The significant increase in total exercise time may support the latter conclusion. The population size that we studied was substantially larger than those of previous crossover studies investigating the effects of inorganic nitrate on exercise or BP behavior, where typically cohorts of sizes n=8 to 15 were sufficient to show effects.^11^, ^21^ Although there was a trend to delay ischemia in the nitrate‐treated group, the increase of exercise capacity in our population may possibly be due to an improvement of skeletal muscle function rather than a direct anti‐ischemic cardiac effect. Improvement of skeletal muscle oxygen handling and mitochondrial efficiency on exercise was previously described in healthy volunteers.^8^, ^9^, ^10^, ^11^, ^12^ Recently the SIRT3‐AMPK‐glut4 activation pathway, which is associated with improved glucose handling in human skeletal muscle, was demonstrated in volunteers with metabolic syndrome.^45^ Coggan et al demonstrated in systolic heart failure patients that a single dose of nitrate‐rich beetroot juice led to a significant increase of skeletal muscle power.^46^ Furthermore, Zamani et al recently reported improved exercise capacity in heart failure patients with preserved ejection fraction.^47^ The effect was mainly due to reduction of vascular resistance and increase of cardiac output on exercise.

In our study population there were no differences in Modified Seattle Questionnaire, use of GTN, or angina between treatment arms. It has to be noted that the latter 3 secondary outcomes were always felt to be particularly weak end points for this particular study design; nevertheless, we felt they should be reported. The treatment arms were short, and angina episodes and GTN use were less frequently reported than expected during the individual test weeks (<1 per treatment period, Table 2).

Nowadays, revascularization is performed rapidly after listing for a procedure. Therefore, we elected a relatively short treatment period (7 to 10 days) to avoid interference with potential clinical revascularization plans. We cannot exclude a greater effect with a longer treatment period.

Many of our patients were elderly, had multivessel disease, and over years adapted their lifestyle to avoid angina. There was a clear discrepancy between their infrequent subjective reporting of angina despite objective severe disease, limiting chest pain on treadmill testing, and electrocardigraphic and echocardiographic evidence of inducible ischemia on functional testing. Even patients who on screening in their own description belonged to Canadian Cardiovascular Society class III were objectively limited on exercise at low workload but reported only 1 or even no angina episodes and little GTN use during the test weeks later. Consequently, the study was underpowered for these secondary end points. Despite previous reports of proangiogenic markers in skeletal muscle (animal model) and plasma in healthy volunteers, we observed no increase in systemic proangiogenic markers in our angina population.

In our study 7 mmol (600 mg) of sodium nitrate was given daily for 1 week. Previously, single doses as low as 3.5 mmol nitrate were effective in lowering blood pressure when given to drug‐naive grade 1 hypertensive volunteers.^30^ A single oral dose of 4 mmol potassium nitrate was sufficient to lower blood pressure in healthy volunteers.^20^ A recent meta‐analysis showed that doses ranging from 300 to 600 mg nitrate (either in the form of beetroot juice or sodium nitrate) suggested a significant moderate benefit on time to exhaustion.^11^ Larsen et al demonstrated in young healthy volunteers that a daily dose of 0.1 mmol/kg given for 3 consecutive days improved mitochondrial efficiency in skeletal muscle.^10^ The dose used in our study is several times higher than an average Western diet intake, which contains ≈100 mg/day.^48^ Our patients ingested their last study capsule ≈2 to 3 hours before their treadmill test. This corresponds to peak plasma nitrite following oral nitrate absorption and enterosalivary bioconversion to nitrite.^19^, ^20^, ^25^ The increases in plasma nitrate and nitrite are similar to those seen in studies demonstrating a blood pressure–lowering effect. Although it is possible that a higher dose might have been more effective, this could also have potentially increased side effects.

We observed gastrointestinal side effects (usually mild) relatively frequently (Table 3). There were no reports of gastrointestinal adverse events in a meta‐analysis of 17 studies assessing nitrate's effects on BP and another meta‐analysis of 16 studies assessing its effects on exercise capacity.^11^, ^21^ However, the majority of these studies were not formal clinical trials of investigational medicinal products requiring reporting of adverse events, and consequently, there might have been underreporting.

Furthermore, most participants in previous studies were young healthy volunteers. Elderly overweight, sedentary patients on polypharmacy are more likely to suffer from chronic esophageal reflux disease or peptic ulcerative disease. In line with this, a significant proportion (38%) of our angina patients were taking proton pump or H_2_ inhibitors. Upper gastrointestinal side effects were well known when much higher doses of inorganic nitrates or nitrites were used for angina treatment at the beginning of the 20th century.^49^

We did not observe the BP‐lowering effect previously observed in healthy young volunteers^19^ and in nonobese drug‐naive grade I hypertensives,^30^ but other studies have suggested that this effect may be absent in obese elderly subjects with insulin resistance.^50^, ^51^ Our study population had end‐organ cardiovascular disease and high BMI (28.6±4.1) kg/m^2^; most were hypertensives and were taking regular background medication.

Another important factor is the polypharmacy these patients were on. Gilchrist et al studied patients with type 2 diabetes mellitus and hypertension (many on antihypertensives), and, unlike with studies in healthy volunteers, they observed no effect of nitrate therapy on the oxygen cost of submaximal exercise.^50^ Our study patients were frequently on antihypertensive treatment (Table 1). In metabolic syndrome, a dysregulation of nitric oxide signaling may interfere with efficacy of some conventional drugs used; on the other hand, certain medications such as statins, angiotensin‐converting enzyme inhibitors, angiotensin receptor blockers, or β blockers may exert their therapeutic effects via modulation of nitric oxide signaling.^52^ Under such circumstances traditional medications may weaken the effect of nitrate supplementation. This may be a reason why other investigators saw more pronounced vasodilator effects even in the elderly population when they chose fairly healthy subjects with no background of antihypertensive drugs. Medications or other underlying medical conditions that alter the gastric pH,^53^, ^54^, ^55^ saliva production, oral bacteria, or intestinal bacterial flora may affect the effectiveness of nitrate treatment. A subgroup analysis of patients who were naive of stomach acid–suppressing medication in our study showed a stronger trend to anti‐ischemic efficacy in our study (see Table 2), supporting the recent concern that antacids may impact on the efficiency of inorganic nitrate–based management in humans^54^, ^55^ by preventing the bioactivation of nitrite to nitrous acid (HNO_2_) in the stomach.^56^

It is difficult to prove antianginal effects in patients on multiple background medications. Although our findings indicate at most modest effects of nitrate when it is given as add‐on therapy, we cannot rule out a stronger benefit if given as a monotherapy. However, we believe that the strength of our study lies in the analysis of real‐life clinical scenarios including patients with multiple comorbidities on polypharmacy. The crossover design made it possible to account for this variation. Chronic angina patients would be very unlikely to be treated with inorganic nitrate monotherapy in the presence of potent first‐line anti‐anginals, and similar studies would be ethically difficult to defend. In this context, it is quite exciting that a dietary treatment strategy has modest benefits on exercise capacity without lowering BP or heart rate. No current standard antianginal medication offers any prognostic benefit for the patients. In the context of a trend to increased time to 1‐mm ST depression and a significantly increased total exercise capacity together with the well‐described prognostic benefit of a nitrate‐rich Mediterranean diet or the fruit‐ and vegetable‐rich DASH diet,^2^, ^48^, ^57^ it appears appropriate to recommend that angina patients adhere to the above diets. Although the treatment period was relatively short (in order to avoid interference of natural disease progression on the outcome analysis in this crossover design), the supplementation resulted in better exercise performance. The results of a longer‐term supplementation will need to be established in further studies. Although rare and usually mild, nausea and vomiting were reported. Patients ought to be alerted to this possible adverse effect.

## Sources of Funding

The study was funded by the Medical Research Council, (UK) grant number G1001536.

## Disclosures

None.

## Supporting information


**Data S1.** Compliance
**Table S1.** Compliance
**Figure S1.** Correlation between the differences of paired treatment arms of total exercise time and time to 1‐mm ST depression (STD).
**Figure S2.** Correlation between the differences of paired treatment arms of total exercise time and time to chest pain onset.
**Figure S3.** Correlation between the differences of paired treatment arms of time to chest pain onset and time to 1‐mm ST depression (STD).
