# Supplementary material for: Inorganic Nitrate in Angina Study: A Randomized Double‐Blind Placebo‐Controlled Trial
Source: J Am Heart Assoc. 2017 Sep 8;6(9):e006478. doi: 10.1161/JAHA.117.006478 (PMC5634294; doi:10.1161/JAHA.117.006478)
Supplement: Supplementary file 1 — Data S1. Compliance Table S1. Compliance Figure S1. Correlation between the differences of paired treatment arms of total exercise time and time to 1‐mm ST depression (STD). Figure S2. Correlation between the differences of paired treatment arms of total exercise time and time to chest pain onset. Figure S3. Correlation between the differences of paired treatment arms of time to chest pain onset and time to 1‐mm ST depression (STD). [file JAH3-6-e006478-s001.pdf]

Supplemental Material

Data S1.

Compliance

The length of capsule treatment was set per protocol to 7-10 days before the test visits to accommodate for patient's non-study commitments, short term appointment changes or weekends which may prohibit the patient to attend. In the vast majority, in 89% (123) of all on-treatment visits the patients were planned to take eight capsules (e.g Tuesday morning to Tuesday morning). In 7% (10) of visits seven capsules, in 3% (4) of visits 9 capsules and in 0.7% (1) of visits 10 capsules were taken prior to the study tests. The compliance with prescribed treatment was very high.

Overall 93.6% of patients took their capsules correctly as prescribed (131 out of 140 treatment periods). On three (2.1%) visits patients missed one capsule intake when counterchecked by the investigator. However on every single occasion patients took their last treatment capsule on the morning visit prior to the tests which was verbally confirmed on their visit.

Table S1. Compliance

**Compliance**<br>*Investigator calculated*		
Correctly completed treatment periods	131	
Missed one capsule*	3	
Took one additional capsule*	1	
Failed to return bottle[£]	3	
Dropped out due to AE	2	(one due to vomiting, one not related to IMP)
Total treatment periods	140	

*in one treatment period (but last capsule was always taken); [£] patient forgot to return the treatment bottle (but they confirmed intake by filling a checklist)

We performed an unblinded post hoc-analysis of the exercise treadmill test data correlating the paired differences (nitrate treatment minus placebo treatment data) between the main treadmill test outcomes against each other using Pearson correlation. There was a strong positive correlation between the time to 1mm ST depression, total exercise time and time to chest pain onset (all p< 0.001):

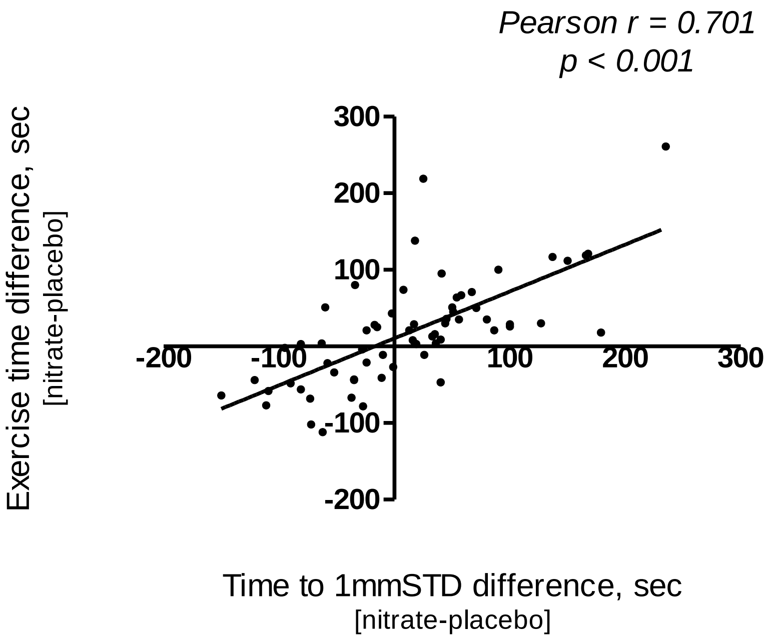

Figure S1. Correlation between the differences of paired treatment arms of total exercise time and time to 1mm ST depression (STD)

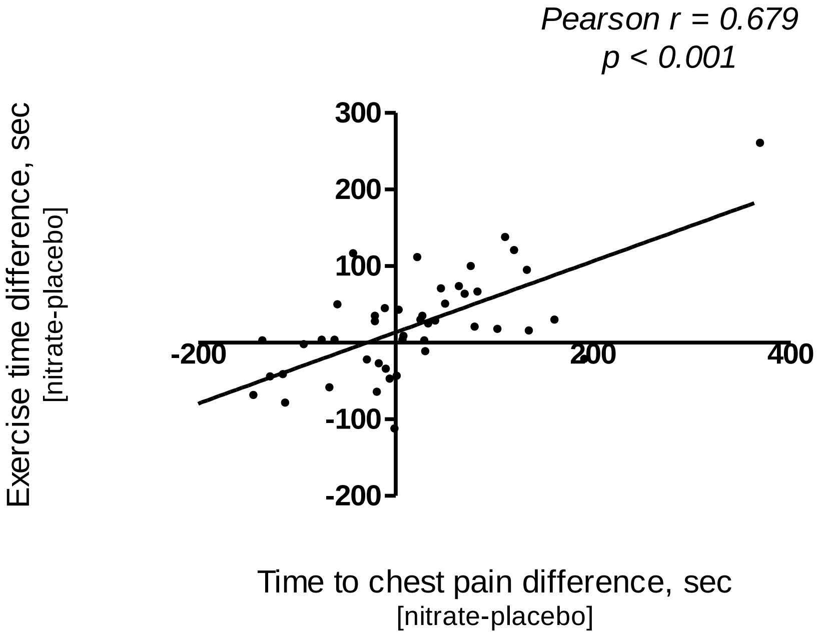

Figure S2. Correlation between the differences of paired treatment arms of total exercise time and time to chest pain onset

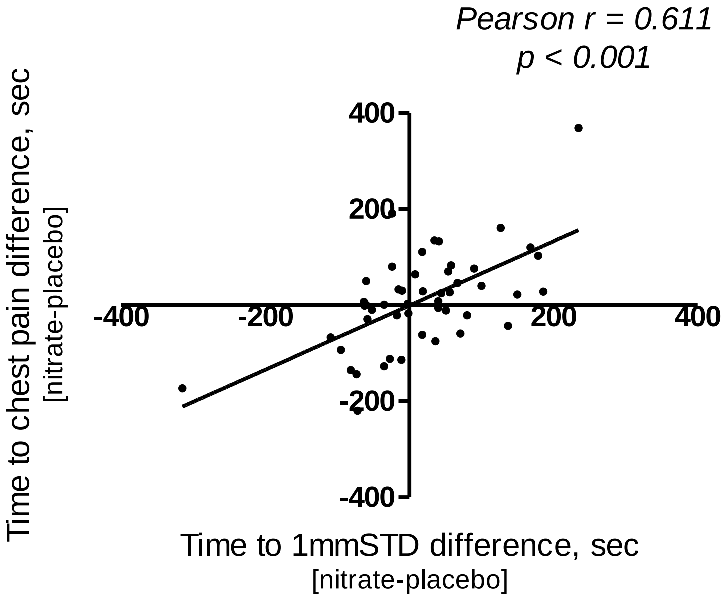

Figure S3. Correlation between the differences of paired treatment arms of time to chest pain onset and time to 1mm ST depression (STD)
